# One-Year Clinical Performance of Injectable and Paste-Type Composite Resins in Non-Carious Cervical Lesions Prepared with Er,Cr:YSGG Laser and Acid Etching: A Randomized Clinical Trial

**DOI:** 10.3390/jfb17020101

**Published:** 2026-02-19

**Authors:** Alperen Değirmenci, Beyza Ünalan Değirmenci

**Affiliations:** 1 Department of Restorative Dentistry, Faculty of Dentistry, Van Yuzuncu Yil University, Van 65080, Türkiye; adegirmenci@yyu.edu.tr; 2 Department of Prosthodontics, Faculty of Dentistry, Van Yuzuncu Yil University, Van 65080, Türkiye

**Keywords:** non-carious cervical lesions, Er,Cr:YSGG laser, injectable composite resin

## Abstract

Background/Objectives: Non-carious cervical lesions (NCCLs) are common defects in adults that can lead to dentin hypersensitivity and aesthetic concerns, for which composite resin restorations currently represent the gold standard of care. However, evidence regarding the long-term clinical superiority of high-filled injectable composites and Er,Cr:YSGG laser-based cavity preparation remains limited. The present study aimed to compare the 1-year clinical performance of two different surface preparation protocols (Er,Cr:YSGG laser vs. conventional bur preparation with phosphoric acid etching) and two composite resin types (high-filled injectable vs. conventional paste-type) in the restoration of NCCLs. Methods: In this prospective, split-mouth, randomized controlled clinical trial, a total of 168 NCCLs in 27 patients were restored. Lesions were randomly allocated to four groups according to the combination of surface preparation (Er,Cr:YSGG laser or phosphoric acid etching) and high-filled injectable composite (G-ænial Universal Injectable) or paste-type composite (G-ænial Anterior). The same universal adhesive system was used in all cases. Clinical evaluations were performed by a blinded examiner at 1 week, 6 months, and 12 months, using the FDI World Dental Federation criteria. Results: At the 1-year follow-up, 25 patients and 150 restorations were available for evaluation, corresponding to a recall rate of 98.22%. High clinical acceptability was observed in all groups with respect to aesthetic, functional, and biological parameters. Retention was 100% in the acid-etched paste-type composite group and ranged from 94.7% to 97.4% in the remaining groups, with no statistically significant differences among groups (*p* > 0.05). A transient increase in postoperative sensitivity was detected in the laser groups at the 1-week evaluation (*p* = 0.026); however, sensitivity scores declined to zero in all groups at 6 months and 1 year. Conclusions: High-filled injectable composites demonstrated 1-year clinical performance comparable to that of conventional paste-type composites in the restoration of NCCLs. Er,Cr: YSGG laser-based cavity conditioning produced outcomes similar to conventional phosphoric acid etching with respect to retention, marginal adaptation, and biological compatibility. The early increase in laser-related postoperative sensitivity was transient and did not compromise long-term clinical success. Taken together, the ease of application and favorable clinical performance of injectable composites indicate that these materials constitute a reliable alternative for the restoration of non-carious cervical lesions.

## 1. Introduction

Non-carious cervical lesions (NCCLs) are pathological defects characterized by a loss of hard dental tissue at the enamel–cementum junction that occurs independently of bacterial caries. Their prevalence in adults has been reported to reach approximately 46.7% and to increase significantly with advancing age [1]. Today, increased life expectancy, the preservation of a greater number of natural teeth, acidic dietary habits, and the growing prevalence of parafunctional habits have led to a higher incidence and greater clinical significance of NCCLs. These factors collectively contribute to a substantial clinical burden, particularly with respect to dentin hypersensitivity, aesthetic concerns, the need for restorative interventions, and potential pulpal and periodontal complications [2,3]. Therefore, elucidating the pathogenesis and optimizing the clinical management of NCCLs, particularly in the context of an aging population, is a priority in contemporary preventive and restorative dentistry [4].

Clinically evident dentin hypersensitivity, aesthetically disturbing cervical defects, progressive loss of tooth structure, subgingival margins that complicate plaque control, and deep or extensive lesions that increase the risk of pulpal exposure or fracture constitute the primary indications for restorative treatment [3,4]. However, the cavity morphology—typically characterized by a wedge-shaped configuration lacking macromechanical retention—the presence of sclerotic dentin, and the location of the cervical/subgingival margins make adhesive bonding and isolation challenging, often reducing long-term restoration survival. When the cervical margin is located in dentin rather than enamel, the reduced presence of enamel prisms and the prevalence of sclerotic dentin hinder hybrid layer formation and render the bonded interface more susceptible to hydrolytic and mechanical degradation. This situation has been associated with increased rates of marginal breakdown, microleakage, and retention loss, thereby underscoring the need for adhesive protocols specifically optimized for dentin-located cervical margins to ensure favorable long-term clinical performance [5,6]. Consequently, both the adhesive protocol and the material properties of the composite resin gain critical importance [7,8].

Composite resins used in conjunction with adhesive systems are widely employed for the restoration of NCCLs, owing to their favorable aesthetic properties, satisfactory mechanical performance, and ability to establish micromechanical bonding with enamel and dentin substrates. [9]. In clinical practice, conventional etch-and-rinse and self-etch adhesive strategies are most frequently employed for bonding in NCCLs [10]. Etch-and-rinse systems generally provide strong enamel bonding following phosphoric acid etching but may be more technique-sensitive when applied to sclerotic dentin [11]. In contrast, self-etch adhesives simplify the clinical procedure and are less dependent on dentin etching, which may be advantageous in deep cervical areas where enamel is limited. Because marginal integrity and retention in NCCLs largely depend on the quality of the adhesive interface, a thorough understanding of the indications and limitations of both etch-and-rinse and self-etch approaches is essential when selecting an adhesive protocol for these lesions [12]. High-filled flowable/injectable composite resins are designed to provide improved flow and cavity adaptation owing to their low viscosity, while their increased filler content aims to enhance mechanical strength and abrasion resistance compared with conventional flowable composites [13,14]. Short- and medium-term clinical trials have reported comparable retention rates and, in many cases, superior marginal adaptation relative to conventional paste-type composite resins [13,15]. However, the number of well-designed, long-term randomized clinical trials that directly demonstrate the superiority of these new-generation materials over conventional paste-type composites remains limited [10].

Cavity surface preparation also plays a critical role in the success of NCCL restorations. In this context, erbium lasers such as Er,Cr:YSGG have been proposed to provide potential clinical advantages in cavity preparation, including minimally invasive removal of hard tissues and favorable modification of the dentin substrate to enhance bonding [16]. Although conventional bur preparation and acid etching aim to enhance micromechanical retention, the formation of a smear layer and the limited effectiveness of etching on sclerotic dentin do not always provide an ideal bonding substrate [4]. Therefore, laser systems such as Er,Cr:YSGG, which provide smear layer-free, irregular, high-energy surfaces, have come to the forefront. Microleakage and bonding performance on Er,Cr:YSGG laser-prepared surfaces have been reported to be comparable to, or in some protocols even superior to, those obtained with conventional bur and acid-etch techniques. However, other investigations have found no clear advantage or have reported increased microleakage when the laser is used as a sole conditioning method [17,18]. Although it has been reported that supplementary acid etching, particularly following laser conditioning, may enhance adhesive infiltration and marginal integrity, the evidence regarding the actual impact of laser-based surface preparation on the long-term retention and marginal adaptation of restorations in NCCLs remains heterogeneous and highly dependent on the specific clinical protocols employed [18,19].

The current literature predominantly examines, separately, the performance of high-fill injectable composites versus paste-type composites and the effectiveness of Er,Cr:YSGG laser roughening compared with conventional bur–acid protocols in the treatment of NCCLs. Randomized split-mouth studies that directly evaluate these two variables concurrently within the same clinical design remain quite limited [15,20]. In this context, the aim of this split-mouth, randomized, controlled clinical trial is to compare the 1-year clinical performance of two different cavity preparation protocols (Er,Cr:YSGG laser roughening and conventional bur preparation followed by phosphoric acid etching) and two different composite resin types (high-filled injectable composite and paste-type composite) in non-carious cervical lesions. Accordingly, the null hypotheses are as follows:

**H**0_1_:  *There is no difference in the 1-year clinical performance of NCCL restorations prepared using Er,Cr:YSGG laser conditioning compared with those prepared using conventional bur–acid etching protocols.*

**H**0_2_:  *There is no difference in the 1-year clinical performance of restorations placed with injectable composite resin versus paste-type composite resin in NCCLs restored under the same cavity preparation protocols.*

## 2. Materials and Methods

### 2.1. Study Design and Ethical Approval

This research was designed as a prospective, split-mouth, randomized, controlled clinical trial with a 1-year clinical follow-up period. The study protocol was approved by the Interventional Ethics Committee of the Faculty of Medicine, Van Yüzüncü Yıl University (decision no. 15, dated 6 August 2019) prior to the commencement of patient enrollment. The research was conducted in accordance with the ethical principles outlined in the 1964 Declaration of Helsinki and its subsequent revisions. The study was reported with reference to the CONSORT guidelines and was registered at ClinicalTrials.gov on 19 September 2019 (registration no. NCT04095520). Before inclusion in the study, all participants were thoroughly informed about the study objectives, treatment procedures, and potential risks, and written informed consent was obtained from all patients.

### 2.2. Participants/Eligibility Criteria

The study included patients over 18 years of age, in the permanent dentition stage, who presented with four or multiples of four non-carious cervical lesions. This requirement ensured that, within the split-mouth design comprising four experimental groups, each patient received one restoration per group, thereby enabling balanced intra-individual comparisons and appropriate random allocation of lesions across the four treatment protocols. Eligible patients had no pulpal or endodontic pathology or percussion sensitivity in the teeth to be treated, had at least one occlusal contact with both antagonist and proximal adjacent teeth, and were expected to attend scheduled follow-up appointments for periodic evaluations.

Individuals were excluded if they presented with systemic conditions that could adversely affect healing or oral health (e.g., uncontrolled diabetes, immunosuppressive disorders, ongoing chemotherapy or radiotherapy, or severe cardiovascular disease); were pregnant, suspected of being pregnant, or breastfeeding; had a known allergy to dental materials; or had diagnosed periodontal disease or poor oral hygiene; were thought to experience excessive occlusal loading on specific teeth due to severe dental crowding; had carious lesions in the cervical region or had previously received desensitizing agents or fluoride treatment in this region; or were undergoing active orthodontic treatment.

In selecting patients with non-carious cervical lesions for inclusion, care was taken to choose wedge-shaped defects located in either the maxilla or mandible, with lesion depth not exceeding 3 mm as measured with a periodontal probe. Lesions classified as grade 4 according to the University of North Carolina index and those presenting sclerotic dentin within the cavity were excluded from the study [21].

### 2.3. Sample Size

The G*Power software (G*Power, version 3.0.10, Düsseldorf, Germany; http://www.psycho.uni-duesseldorf.de/aap/projects/gpower) (accessed on 10 July 2019) was used to calculate the required sample size. To detect differences between the study groups, a power analysis was performed assuming a moderate effect size (f = 0.25), 80% statistical power (1−β = 0.80), and a 5% significance level (α = 0.05). Based on these parameters, it was determined that a minimum of 35 restorations per group, and 140 restorations in total, would be sufficient.

To compensate for potential dropouts during the follow-up period and to preserve the planned statistical power, a larger number of lesions was initially evaluated, and the predefined inclusion and exclusion criteria were applied. Following this evaluation, a total of 168 restorations of non-carious cervical lesions in 27 patients were deemed eligible and included in the study, and all clinical assessments were performed on these restorations. Thus, the initially calculated minimum sample size was achieved, and the final sample was further strengthened by accounting for possible losses to follow-up.

### 2.4. Randomization and Blinding

After enrollment, patients and their non-carious cervical lesions were randomly allocated to one of four experimental groups according to a split-mouth design. Randomization was performed using a random number table generated with the Research Randomizer Program (http://www.randomizer.org/form.htm) (accessed on 25 January 2021) [22]. This method ensured a balanced allocation of lesions in each patient’s mouth to one of four treatment groups, which were defined according to the cavity preparation protocol (acid etching or laser etching) and the type of restorative material used (injectable or paste-type composite).

In the blinding procedure, clinical evaluations of the restorations were performed by a single examiner who was not involved in the restorative procedures and was blinded to the group assignments. Furthermore, participants were not informed about which etching protocol or type of composite material had been used for each tooth. Thus, a double-blind evaluation was ensured, as neither the patients nor the clinical examiner had any knowledge of the specific restorative material or surface preparation protocol applied to the individual teeth.

### 2.5. Clinical Application Protocol

#### 2.5.1. Cavity Preparation

Before initiating the restorative procedures, all patients received oral hygiene instruction. Subsequently, all teeth scheduled for treatment were cleaned with pumice and rubber cups to remove the salivary pellicle and dental plaque, and no local anesthesia was administered during the procedure.

In the acid-etching group, a conservative enamel bevel was prepared along the cervical enamel margin and 34.5% phosphoric acid gel (Vococid^®^, VOCO GmbH, Cuxhaven, Germany) was applied to the enamel and dentin surfaces for 20 s. This was followed by rinsing each tooth with pressurized water for at least 15 s and gentle air-drying. Cotton rolls and a saliva ejector were used to prevent salivary contamination, and the etched surfaces were examined for the presence of a characteristic chalky-white appearance.

**Figure 1:**
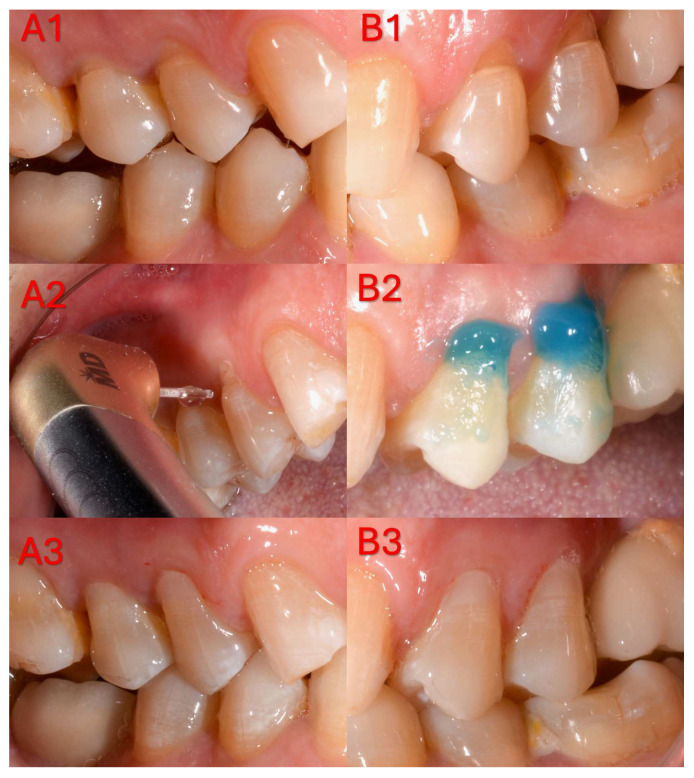
Clinical stages of a representative non-carious cervical lesion restored using two different surface conditioning protocols. (**A**) Laser-etched group: (**A1**) baseline clinical photograph before treatment; (**A2**) cavity surface conditioning performed with Er,Cr: YSGG laser irradiation; (**A3**) final appearance after composite resin restoration. (**B**) Acid-etched group: (**B1**) baseline clinical photograph before treatment; (**B2**) enamel and dentin etching with 34.5% phosphoric acid gel; (**B3**) final appearance after composite resin restoration.

In the laser group, cavity roughening was performed using an Er,Cr:YSGG hard tissue laser (WaterLase^®^ iPlus, Biolase Technologies Inc., Santa Ana, CA, USA) with a wavelength of 2.78 µm. The procedure was carried out with a 600-µm-diameter sapphire tip (MGG6–6 mm) and a gold handpiece, at 2.25 W output power, 50 Hz repetition rate, 140 µs pulse duration, and 60% air and 30% water cooling. The optical tip was moved along the cavity surface at a right angle and within the working distance recommended by the manufacturer for approximately 10–15 s, without contacting the tooth structure [23]. Each pulse delivered 45 mJ of energy, corresponding to an energy density of approximately 15.91 J/cm^2^. To prevent contamination of the tip during the procedure, it was periodically cleaned with alcohol-soaked gauze, and laser irradiation was discontinued once the surface exhibited an opaque white appearance (Figure 1).

#### 2.5.2. Adhesives and Restoration

In all cavities, a single-bottle, single-step adhesive procedure was standardized by applying a universal adhesive agent (G-Premio BOND, GC Dental Products Corp, Aichi, Japan) in accordance with the manufacturer’s instructions. Subsequently, non-carious cervical lesions were restored with either a microhybrid conventional paste-type composite (G-ænial Anterior, GC Dental Products Corp, Aichi, Japan) or a high-filled injectable composite resin (G-ænial Universal Injectable, GC Dental Products Corp, Aichi, Japan). The materials were placed in a single increment, following the manufacturer’s recommendations, and adapted carefully along the cavity walls to prevent void or bubble formation. The type, composition, filler content, and lot numbers of the restorative and adhesive materials used are detailed in Table 1.

Composite resins were polymerized using an LED curing unit (Elipar S10, 3M ESPE, St. Paul, MN, USA) emitting light in the 430–480 nm wavelength range, at an irradiance of approximately 1200 mW/cm^2^. For each restoration, the light guide was positioned perpendicular to the composite surface and activated for the exposure time recommended by the manufacturer. The output intensity of the curing unit was checked with an LED radiometer (LED Radiometer, SDI Dental Limited, Victoria, Australia) before each use to ensure that it remained within the standard operating range.

Finishing and polishing procedures were completed in the same session in which the restorations were placed. For this purpose, multi-step disc systems (Sof-Lex, 3M ESPE Dental Products, St. Paul, MN, USA), diamond-impregnated two-step flexible discs (CLEARFIL Twist DIA, EVE Ernst Vetter GmbH, Keltern, Germany), and a two-step disc–rubber system (Enhance^®^ and PoGo^®^, Dentsply DeTrey GmbH, Konstanz, Germany) were used. The abrasive content, particle size distribution, and lot numbers of the polishing systems employed are presented in Table 2. Following restoration, all patients received brief counseling regarding potential etiological factors associated with NCCLs and were advised on general measures to minimize mechanical and chemical stresses on the cervical tooth structure.

### 2.6. Clinical Evaluation (FDI Criteria)

Clinical check-ups were conducted at three time points—baseline (week 1), 6 months, and 12 months—and at each visit the clinical evaluation criteria of the FDI World Dental Federation were applied (Table A1 in Appendix A) [24]. Based on these criteria, the restorations were evaluated under three main domains: aesthetic, functional, and biological. The aesthetic evaluation considered surface gloss, surface discoloration, color stability, translucency, and anatomical form. The functional evaluation focused on fractures and retention, marginal adaptation, wear, and the patient’s satisfaction with the restoration. The biological evaluation encompassed post-operative sensitivity, recurrent caries, erosion/abfraction, tooth integrity, periodontal response, adjacent mucosa, and oral and general health status. Each restoration was scored on the FDI scale from 1 to 5 within these subdomains; scores of 1 to 3 were regarded as clinically acceptable, whereas scores of 4 or 5 were classified as clinically unacceptable for the respective criterion. The clinical acceptability rate (CAR) was calculated as the percentage of restorations exhibiting FDI scores between 1 and 3 for the respective parameter at each recall visit.

### 2.7. Statistical Analysis

All statistical analyses were performed using IBM SPSS Statistics software (version 23.0, IBM Corp., Armonk, NY, USA). The Fleiss kappa test was employed to evaluate the agreement of measurements over time. Pearson’s chi-square test was used for intergroup comparisons of categorical variables; when expected cell counts were insufficient, Fisher’s exact test was applied, and Yates’ continuity correction was used when appropriate. Survival analysis was conducted using the Kaplan–Meier method, and differences between survival curves were evaluated with the log-rank test. For the purposes of this analysis, failure was defined as complete loss of the restoration (failure = score 5 according to the FDI “Fractures and Retention” criterion). Categorical variables were summarized as frequency (percentage). A *p*-value of <0.05 was considered to indicate statistical significance.

## 3. Results

**Figure 2:**
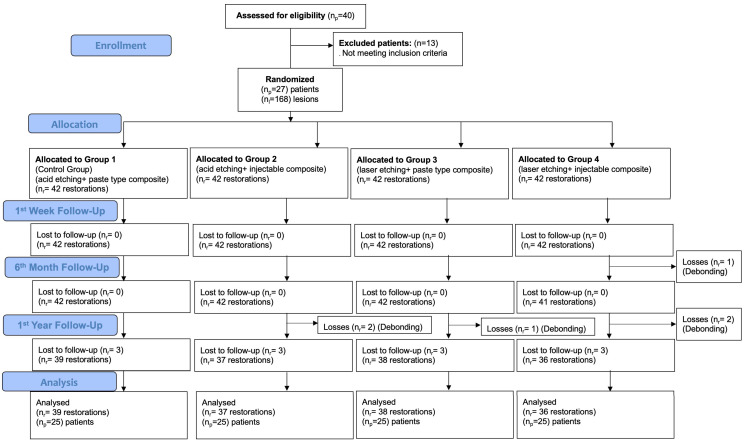
Flowchart of the study.

Initially, a total of 27 patients and 168 non-carious cervical lesions (NCCLs) were restored in the study. After the one-year follow-up period, 25 patients attended their recall appointments, and 150 restorations were clinically evaluated. Accordingly, the patient recall rate was calculated as 98.22% (Figure 2). At the one-year follow-up, two patients discontinued participation in the study due to relocation to other cities.

Table 3 presents the distribution of restored NCCLs according to dental arch, tooth type, etching method, and composite material. In the maxillary arch, the majority of the 51 lesions restored following acid etching were located in premolar and incisor teeth, whereas in the mandibular arch, a substantial proportion of the 33 lesions was situated in the premolar region. Similarly, the 84 lesions treated with laser etching were evenly distributed among canines, premolars, and incisors in both the maxillary and mandibular arches. At baseline, no statistically significant differences were observed between the groups with respect to the distribution of the teeth included or the dental arch in which they were located (*p* > 0.05).

**Figure 3:**
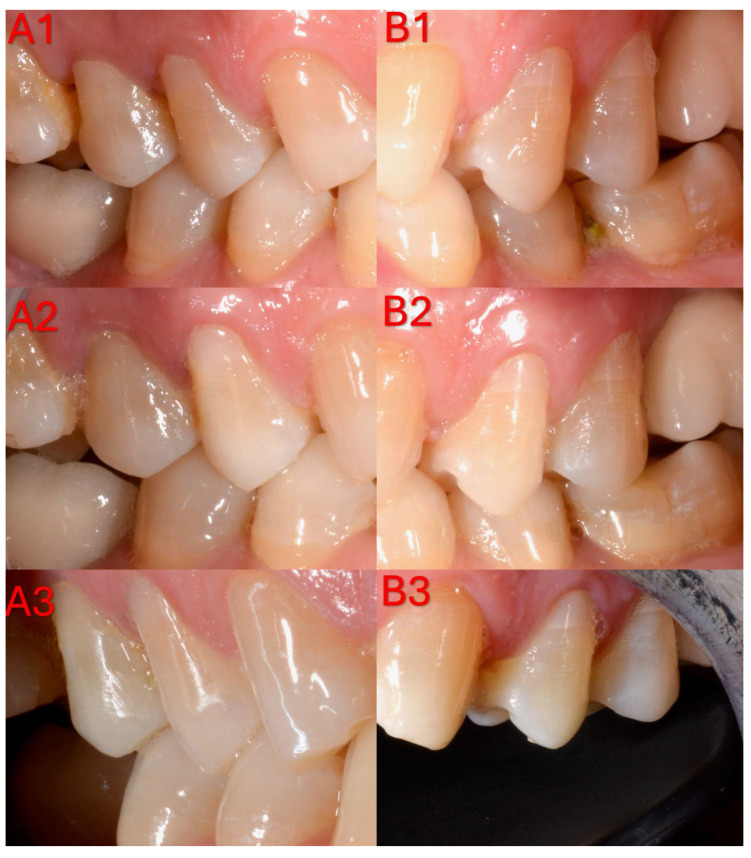
Representative clinical follow-up photographs of non-carious cervical lesion restorations evaluated according to the FDI World Dental Federation criteria at different time points. (**A**) Laser-etched group: (**A1**), 1-week recall (baseline); (**A2**), 6-month follow-up; (**A3**), 12-month follow-up. (**B**) Acid-etched group: (**B1**), 1-week recall (baseline); (**B2**), 6-month follow-up; (**B3**), 12-month follow-up.

According to the FDI criteria, high clinical acceptability rates were obtained in all groups over the 1-year follow-up period with respect to aesthetic, functional, and biological parameters (Table 4, Table A4 and Table A5) (Figure 3).

Examination of the aesthetic criteria revealed that clinically acceptable scores (1–2) predominated in all groups for surface gloss, surface staining, color stability/translucency, and anatomical form, and that 100% clinical acceptability was maintained at the 1-week, 6-month, and 1-year evaluations. Although the frequency of score 2 increased over time, these changes were not statistically significant (*p* > 0.05).

With respect to fractures and retention, regarded as the most critical parameters within the functional criteria, the retention rate in the paste-type composite group following acid etching was 100% at all evaluation time points. After 1 year of follow-up, the retention rates were 94.8% in the injectable composite group following acid etching, 94.73% in the injectable composite group following laser etching, and 97.4% in the paste-type composite group following laser etching. Despite these losses, no statistically significant differences in retention were detected among the groups at 1 week, 6 months, or 1 year (*p* = 1.00, *p* = 0.62, *p* = 0.74, respectively). Although an increase in score 2 was observed over time for marginal adaptation and wear, these changes were not statistically significant (*p* = 0.362).

Clinically acceptable scores were maintained in all groups throughout the 1-year follow-up period with respect to the biological criteria (post-operative sensitivity, recurrent caries, tooth integrity, periodontal response, adjacent mucosa, and oral/general health), yielding a clinical acceptability rate of 100%. A transient increase in post-operative sensitivity scores was observed at the 1-week follow-up only in the laser-etching groups (*p* = 0.026); however, sensitivity scores decreased to 0 in all groups at the 6-month and 1-year evaluations. These findings indicate that, according to the FDI criteria, both composite types and both surface-conditioning methods demonstrated generally comparable and successful clinical performance over the 1-year observation period (Table 4).

**Figure 4:**
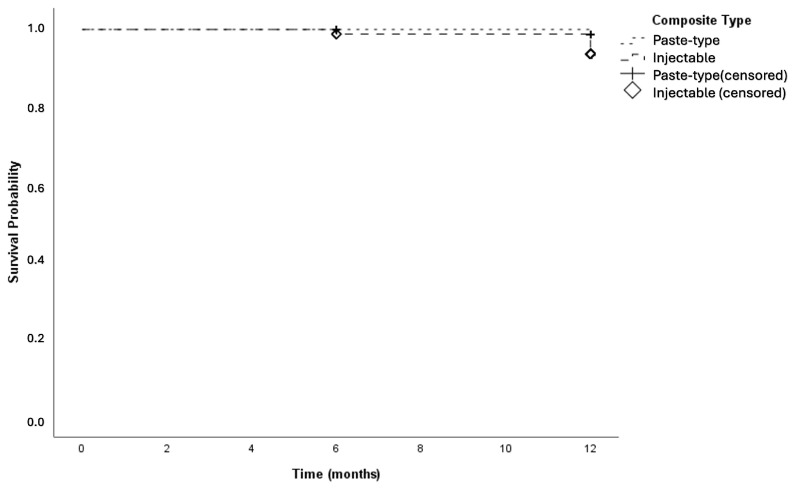
Kaplan–Meier survival curves of restorations according to composite type.

**Figure 5:**
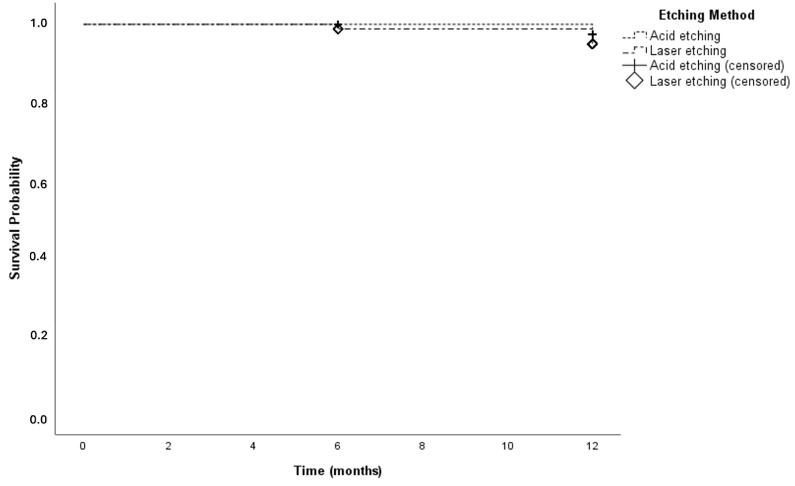
Kaplan–Meier survival analysis based on the etching protocol.

Kaplan–Meier survival analysis demonstrated 12-month cumulative survival rates of 98.7% for the paste-type composite and 93.7% for the high-filled injectable composite, with no statistically significant difference observed between the two composite materials (*p* = 0.096) (Figure 4). Similarly, for the cavity preparation methods, the 12-month cumulative survival rates were 97.4% in the acid-etching group and 95.0% in the Er,Cr:YSGG laser-etched group with no statistically significant difference in survival (*p* = 0.402) (Figure 5).

## 4. Discussion

Evaluating the restorative success of NCCLs using multidimensional criteria—such as retention, marginal adaptation, and biological compatibility—is regarded as fundamental for the objective assessment of clinical performance [25]. The findings of the present study demonstrated that the 1-year clinical performance of the high-filled injectable composite and the conventional paste-type composite, when applied under different surface preparation protocols, was comparable, and that both materials achieved high success according to the FDI criteria. With respect to retention, statistically similar outcomes were obtained, with a 100% retention rate in the paste-type composite group following acid etching and rates ranging from 94.7% to 97.4% in the remaining groups (*p* > 0.05). The observation that changes in the aesthetic and functional sub-criteria remained within clinically acceptable limits supports the acceptance of the null hypotheses for both the surface preparation method (H0_1_) and the composite type (H0_2_). Nonetheless, the transient increase in post-operative sensitivity observed at the 1-week follow-up in the laser-etching groups (*p* = 0.026) represents a noteworthy exception with respect to the biological criteria.

Several factors are considered critical for retention success, including the reduced etchability of sclerotic dentin [2], which complicates hybrid layer formation, and the increase in stress accumulation in the cervical region due to occlusal loading, thereby challenging the bonding interface [3]. Indeed, randomized clinical trials employing universal adhesives have demonstrated that the degree of dentinal sclerosis and the internal angle morphology of the lesion can increase the risk of retention loss [26], whereas occlusal wear and the presence of antagonistic teeth may contribute to the etiopathogenesis and functional load profile of NCCLs [12]. The 1-year retention rates observed in the present study, when compared with the first-year data reported by Peumans et al.—who documented retention rates of approximately 86–87% at 3 years in NCCLs restored using two different application modes of a universal adhesive—similarly reflect a pattern of high early retention and a limited number of lost restorations [12]. Moreover, a similar trend was observed in the multimodal adhesive study by Manarte-Monteiro et al., which reported an overall 1-year retention rate of 97.5% across different adhesive application modes, thereby corroborating that composite-based NCCL restorations generally remain stable in the short term despite variations in adhesive protocols and materials [27]. Similarly, a study comparing pre-heated and non-heated composites reported retention rates of 90–97% over a period of up to 24 months, further supporting the short-term clinical success of composite-based NCCL restorations despite variations in restorative technique or material viscosity [28]. In recent years, numerous clinical studies and systematic reviews have reported survival or retention rates generally exceeding 80% for composite-based NCCL restorations over 2–5 years of follow-up, despite variability in universal adhesive formulations, application times, and cavity preparation protocols, thereby underscoring that the adhesive protocol is a more critical determinant of clinical outcome than the material type [29,30,31]. However, in the 7.7-year follow-up study by Lührs et al., the overall retention rate was reported as 82.8%, whereas retention loss increased to 27.8% in lesions that were merely cleaned without any cavity preparation, indicating that there are also studies in the literature reporting comparatively lower retention rates [32]. This discrepancy is thought to be attributable to factors such as the increasing prominence of fatigue and hydrolytic degradation at the adhesive interface over longer follow-up periods, as well as the adverse effect of omitting selective roughening on highly sclerotic dentin surfaces on the quality of the hybrid layer.

The favorable clinical performance of injectable composites observed in the present study can be attributed to the balanced combination of superior cavity adaptation, resulting from their low viscosity, and the mechanical stability conferred by their high filler content. High-filled flowable/injectable materials, owing to their thixotropic behavior, effectively wet cavity walls—even in cavities with limited macromechanical retention and irregular surfaces—thereby reducing the formation of voids and bubbles and consequently enhancing marginal adaptation. Consistent with these findings, Hançer Sarıca et al. reported that G-ænial Universal Injectable provided superior adaptation to cavity walls and margins in Class II restorations and reduced the risk of void formation in challenging areas [33]. Similarly, Basheer et al. demonstrated that in high-filled flowable composites such as G-ænial Universal FLO and G-ænial Universal Injectable, a lower elastic modulus and increased flowability significantly reduce microleakage by decreasing polymerization shrinkage stresses [34]. Another study demonstrated that resin composites with reduced viscosity, when applied using an injection technique, produced fewer interfacial defects at the gingival wall compared with the placement of the same material using a spatula, thereby highlighting that the combination of injection and low viscosity enhances internal adaptation [35]. These findings suggest that the favorable marginal adaptation and high retention rates observed in NCCL restorations over the 1-year follow-up in the present study are consistent with the rheological properties of the injectable composite. Conversely, the absence of significant differences between the injectable composite and the conventional paste-type composite with respect to retention, fracture, wear, and anatomical form can be attributed to the fact that both materials exhibit similar mechanical properties due to their high filler content. The literature indicates a strong correlation between the inorganic filler fraction of composite systems and their elastic modulus and flexural strength, and it has been reported that high-filled flowable/nanohybrid composites can demonstrate mechanical performance equivalent or comparable to that of certain hybrid paste composites [36]. In a 2-year Class II clinical trial, Hançer Sarıca et al. compared G-ænial Universal Injectable with conventional and bulk-fill composites and found no significant differences among the materials for most FDI criteria, attributing these findings to their high filler content and consequent similarity in mechanical behavior [33]. Consistent with these findings, the principal advantage of the injectable composite in the present study was observed in terms of cavity adaptation and ease of application, whereas the absence of significant differences in mechanically oriented outcomes—such as retention and fracture—when compared with a paste-type composite of similar filler content can be regarded as an expected result.

In the present study, the higher level of postoperative sensitivity observed at the first-week evaluation following laser roughening can be attributed to both pulpal thermal effects and the morphological alterations induced by the laser on dentinal tubules. In vitro investigations employing hard tissue lasers (particularly Er:YAG, Er,Cr:YSGG, and high-power diode lasers) have shown that, under certain parameters, these devices can produce melting, recrystallization, and narrowing or obliteration of tubule orifices on the dentin surface, in addition to a short-term temperature increase and a limited pulpal inflammatory response [37]. Meng et al. reported that TNF-α and HSP-70 expression increased significantly on day 1 following 980-nm diode laser application, that inflammation gradually subsided between days 7 and 14, and that the intrapulpal temperature rise remained below the critical threshold [38]. These findings suggest that early pulpal inflammation and increased neural excitability may manifest clinically as heightened sensitivity during the first week. Conversely, it has been reported that hard tissue lasers, rather than completely occluding dentinal tubules, can under certain parameters expose tubule orifices, increase surface roughness, or produce only partial occlusion; such effects may initially facilitate the hydrodynamic mechanism and thereby exacerbate dentinal sensitivity [39]. Dündar et al. also reported that Er,Cr:YSGG laser irradiation significantly modifies the dentin surface by altering tubule patency and surface roughness, noting that this effect may initially be unfavorable with respect to sensitivity but may, in the longer term, promote tubule obliteration and thus yield more favorable outcomes [40]. In the present study, the reduction in sensitivity observed over time in the laser group, in line with the aforementioned literature, can be attributed to the resolution of the initial, limited thermal and inflammatory pulpal response, the maturation of laser-induced tubule plugs, and the formation of secondary or tertiary dentin, collectively resulting in decreased dentin permeability.

The finding that marginal adaptation values in the laser-prepared cavity group were comparable to those obtained with conventional methods appears consistent with the wetting and adaptation advantages of injectable composites arising from their low viscosity. Low viscosity facilitates superior wetting of the cavity walls, particularly in the cervical region, and enables the material to spread along the walls without leaving voids; this, in turn, can be associated with higher initial proportions of “continuous margins” in the restorations [41]. Low-viscosity resins used in cervical cavities have been reported to demonstrate high marginal adaptation and retention rates over 2–3 years of follow-up [32]. Both clinical and laboratory studies have indicated that, in NCCL models, a thin layer of low-viscosity resin can function as a “stress-absorbing interlayer,” distributing polymerization shrinkage stresses and reducing marginal gaps, particularly at the gingival margin [42]. The favorable marginal integrity observed in the laser group in the present study is consistent with this stress-distributing effect and supports the notion that injectable composites can be safely used on laser-prepared surfaces. However, it has also been shown that bulk-fill materials placed in large volumes in a single increment are associated with increased overall polymerization shrinkage and more pronounced marginal deterioration after aging, although differences among various placement techniques tend to diminish following thermomechanical loading [43]. The minimal intergroup differences in marginal adaptation observed during long-term follow-up in the present study suggest that, rather than conferring a permanent standalone advantage, the injectable composite provides clinically adequate and predictable marginal integrity when used with an appropriate layering protocol.

The literature indicates that enamel and dentin surfaces prepared with erbium lasers exhibit a smear-free, high-energy morphology conducive to micromechanical retention; when combined with appropriate adhesive protocols, these surfaces can provide bond strengths comparable to conventional acid etching and yield clinically acceptable outcomes with respect to marginal adaptation and marginal discoloration [44]. Moreover, laser-based cavity preparation has been reported to yield outcomes that are at least comparable to, and in some cases superior to, conventional bur preparation with respect to marginal adaptation and color stability in Class V restorations [19]. The stable marginal parameters observed in the laser group in the present study are consistent with these reports and support the conclusion that the combination of laser-prepared surfaces with injectable composites represents a clinically safe and predictable option with respect to marginal integrity and color stability.

Several limitations should be taken into account when interpreting the findings of the present study. First, although care was taken to allocate restorations in multiples of four per patient, restorations were not assigned to predefined quadrants or jaws. Therefore, perfect split-mouth standardization at the quadrant level could not be achieved. Also the 1-year follow-up period is insufficient to fully characterize the long-term clinical behavior of parameters that may exhibit progressive changes over time, such as marginal adaptation and color stability. In addition, the single-center design limits the generalizability of the results to other clinical environments and more heterogeneous patient populations. Future investigations with larger sample sizes and multicenter designs, incorporating follow-up periods of 3–5 years, are warranted to more comprehensively elucidate both the long-term performance of laser-etched surfaces and the clinical behavior of next-generation injectable composites in patients with varying risk profiles.

## 5. Conclusions

Based on the 1-year follow-up results of this randomized controlled clinical trial, the following conclusions can be drawn:

1. High-filled injectable composites demonstrated clinical performance comparable to that of conventional paste-type composites in the restoration of non-carious cervical lesions.
2. The cavity roughening protocol performed with an Er,Cr:YSGG laser produced outcomes comparable to those achieved with conventional phosphoric acid etching, with similar 1-year retention rates, marginal adaptation scores, and overall FDI-based clinical acceptability in NCCL restorations.
3. The increase in post-operative sensitivity observed in the laser-roughening groups during the first week was transient and did not adversely affect long-term clinical success.

## Figures and Tables

**Table 1:** Restorative and adhesive materials used in this study.

Material	Manufacturer	Type	Composition	% in Weight	LOT Number
G-ænial Anterior	GC Dental Products Corp, Aichi, Japan	microhybrid conventional composite resin	Matrix: Methacrylate monomers Fillers: Silica, Strontium glass, Lanthanoid fluoride	Matrix: 24% Fillers: 76%	190327A 190627A
G-aenial Universal Injectable	GC Dental Products Corp, Aichi, Japan	injectable composite resin	Matrix: Methacrylate monomer Fillers: Silica, Barium glass	Matrix: 31% Fillers: 69%	190518A 190410D
G-Premio BOND	GC Dental Products Corp, Aichi, Japan	universal adhesive	TEGDMA, MDP, 4-MET, MDTP, acetone, water, initiator, silica		1901242
Vococid^®^	VOCO GmbH, Cuxhaven, Germany	phosphoric acid	%34.5 phosphoric acid		1906566

TEGDMA: Triethylene Glycol Dimethacrylate. MDP: 10-methacryloyloxydecyl dihydrogen phosphate. 4-MET: 4-[2-(methacryloyloxy)ethoxycarbonyl] phthalic acid. MDTP: 10-methacryloyloxydecyl dihydrogen thiophosphate methacrylate monomer.

**Table 2:** Polishing system used in this study.

Polishing System	Manufacturer	Polishing Procedure	Abrasive Content	Particle Size	LOT Number
Sof-Lex	3M ESPE Dental Products, St. Paul, MN, USA	Multi-step rubber polishing discs	Aluminum oxide	Course: 60 μm Medium: 29 μm Fine: 14 μm Extra fine: 5 μm	N958970
CLEARFIL Twist DIA	EVE Ernst Vetter GmbH, Keltern, Germany	Two-step flexible polishing discs	Diamond	First step: Rubber disc for smoothing Second step: Rubber disc for polishing	434877
Enhance^®^ and PoGo^®^	Dentsply DeTrey GmbH, Konstanz, Germany	Two-step polishing system	Aluminum oxide, diamond abrasive	Enhance: 40 μm Pogo: 7 μm	131014

**Table 3:** Distribution of NCCLs restored in the study.

Cavity Preparation Protocols	Dental Arch	Tooth	Composite Type		Total	*p*
			High-Filled Injectable Composite	Paste-Type Composite		
Conventional Bur–Acid Etching (*n* = 84)	Maxillary (*n* = 51)	Canine	4	3	7	0.914
		Incisive Teeth	7	6	13	
		Molar	1	2	3	
		Premolar	15	13	28	
	Mandibular (*n* = 33)	Canine	1	2	3	0.584
		Incisive Teeth	2	4	6	
		Molar	1	3	4	
		Premolar	11	9	20	
Er,Cr:YSGG laser (*n* = 84)	Maxillary (*n* = 37)	Canine	7	4	11	0.542
		Incisive Teeth	3	5	8	
		Molar	1	0	1	
		Premolar	9	8	17	
	Mandibular (*n* = 47)	Canine	3	2	5	0.656
		Incisive Teeth	6	4	10	
		Molar	1	1	2	
		Premolar	12	18	30	

**Table 4:** Distributions of values measured at different times depending on the etching method and composite used.

			Injectable Composite			CAR	Paste-Type Composite			CAR
			1 Week	6 Months	1 Year		1 Week	6 Months	1 Year	
			(1/2/3/4/5)	(1/2/3/4/5)	(1/2/3/4/5)		(1/2/3/4/5)	(1/2/3/4/5)	(1/2/3/4/5)	
Acid-Etching	Functional Properties	Fractures and Retention	42/0/0/0/0	42/0/0/0/0	37/0/0/0/2	94.8%	42/0/0/0/0	42/0/0/0/0	38/1/0/0/0	100%
		Marginal Adaptation	42/0/0/0/0	42/0/0/0/0	34/3/0/0/0	100%	42/0/0/0/0	42/0/0/0/0	35/4/0/0/0	100%
	Biological Properties	Postoperative Sensitivity	42/0/0/0/0	42/0/0/0/0	37/0/0/0/0	100%	42/0/0/0/0	42/0/0/0/0	39/0/0/0/0	100%
Er,Cr:YSGG laser	Functional Properties	Fractures and Retention	42/0/0/0/0	41/0/0/0/1	36/0/0/0/2	94.7%	42/0/0/0/0	42/0/0/0/0	38/0/0/0/1	97.4%
		Marginal Adaptation	42/0/0/0/0	41/0/0/0/0	35/1/0/0/0	100%	42/0/0/0/0	42/0/0/0/0	33/5/0/0/0	100%
	Biological Properties	Postoperative Sensitivity	36/6/0/0/0	41/0/0/0/0	36/0/0/0/0	100%	34/8/0/0/0	42/0/0/0/0	38/0/0/0/0	100%

CAR: Clinical acceptability rate.

## Data Availability

Data are contained within the article.

## Appendix A

**Table A1:** FDI Criteria and Scoring System for Esthetic Properties [24].

Scores	1. Surface Gloss	2. Surface Staining	3. Color Stability and Translucency	4. Anatomic Form
1. Clinically excellent/very good	1.1 Lustre comparable to enamel.	2a.1 No surface staining. 2b.1 No marginal staining.	3.1 Good color match, no difference in shade and/or translucency.	4.1 Form is ideal.
2. Clinically good (after polishing probably very good)	1.2.1 Slightly dull, not noticeable from speaking distance. 1.2.2 Some isolated pores.	2a.2 Minor surface staining, easily removable by polishing. 2b.2 Minor marginal staining, easily removable by polishing.	3.2 Minor deviations in shade and/or translucency.	4.2 Form is only slightly deviated from normal.
3. Clinically sufficient/satisfactory	1.3.1 Dull surface but acceptable if covered with film of saliva. 1.3.2 Multiple pores on more than one third of the surface.	2a.3 Moderate surface staining that may also present on other teeth, not esthetically unacceptable. 2b.3 Moderate marginal staining, not esthetically unacceptable.	3.3 Distinct deviation but acceptable. Does not affect esthetics: 3.3.1 More opaque 3.3.2 More translucent 3.3.3 Darker 3.3.4 Brighter.	4.3 Form deviates from normal but is esthetically acceptable.
4. Clinically unsatisfactory (but reparable)	1.4.1 Rough surface cannot be masked by saliva film; simple polishing is not sufficient. Further intervention necessary. 1.4.2 Voids.	2a.4 Unacceptable surface staining; major intervention necessary for improvement. 2b.4 Pronounced marginal staining; major intervention necessary for improvement.	3.4 Localized clinical deviation that can be corrected by repair: 3.4.1 Too opaque 3.4.2 Too translucent 3.4.3 Too dark 3.4.4 Too bright.	4.4 Form is affected and esthetically unacceptable. Intervention/correction is necessary.
5. Clinically poor (replacement necessary)	1.5 Very rough, unacceptable plaque-retentive surface.	2a.5 Severe surface and/or subsurface staining, not accessible for intervention. 2b.5 Deep marginal staining, not accessible for intervention.	3.5 Unacceptable. Replacement necessary.	4.5 Form is unsatisfactory and/or lost. Repair not feasible/reasonable. Replacement needed.

**Table A2:** FDI Criteria and Scoring System for Functional Properties [24].

Scores	5. Fractures and Retention	6. Marginal Adaptation	7. Wear	10. Patient’s View
1. Clinically excellent/very good	5.1 No fractures/cracks.	6.1 Harmonious outline, no gaps, no white or discolored lines.	7a.1 Physiological wear equivalent to enamel. 7b.1 Wear corresponding to 80–120% of enamel.	10.1 Entirely satisfied with esthetics and function.
2. Clinically good (after polishing probably very good)	5.2 Small hairline crack.	6.2.1 Marginal gap (<150 μm), white lines. 6.2.2 Small marginal fracture removable by polishing. 6.2.3 Slight ditching, slight steps/flashes, minor irregularities.	7a.2 Normal wear only slightly different from enamel. 7b.2 50–80% or 120–150% wear compared to corresponding enamel.	10.2 Satisfied. 10.2.1 Esthetics. 10.2.2 Function (e.g., minor roughness).
3. Clinically sufficient/satisfactory	5.3 Two or more/larger hairline cracks and/or material chip fracture not affecting marginal integrity or approximal contact.	6.3.1 Gap < 250 μm not removable. 6.3.2 Several small marginal fractures. 6.3.3 Major irregularities, ditching, flashes, steps.	7a.3 Wear rate different from enamel but within biological variation. 7b.3 <50% or 150–300% of corresponding enamel.	10.3 Minor criticism but no adverse clinical effects. 10.3.1 Esthetic shortcomings. 10.3.2 Some lack of chewing comfort. 10.3.3 Unpleasant treatment procedure.
4. Clinically unsatisfactory (but reparable)	5.4.1 Material chip fractures damaging marginal quality or approximal contacts. 5.4.2 Bulk fractures with partial loss (<50% of restoration).	6.4.1 Gap > 250 μm or dentine/base exposed. 6.4.2 Severe ditching or marginal fractures. 6.4.3 Larger irregularities or steps (repair necessary).	7a.4 Wear considerably exceeds normal enamel wear or occlusal contact points are lost. 7b.4 Restoration > 300% of enamel wear or antagonist > 300%.	10.4 Desire for improvement. 10.4.1 Esthetics. 10.4.2 Function (e.g., tongue irritation). Reshaping or refurbishing possible.
5. Clinically poor (replacement necessary)	5.5 Partial or complete loss of restoration or multiple fractures.	6.5.1 Restoration (complete or partial) is loose but in situ. 6.5.2 Generalized major gaps or irregularities.	7a.5 Wear is excessive. 7b.5 Restoration or antagonist >500% of corresponding enamel.	10.5 Completely dissatisfied and/or adverse effects, including pain.

**Table A3:** FDI Criteria and Scoring System for Biological Properties [24].

Scores	11. Postoperative (Hyper-)Sensitivity and Tooth Vitality	12. Recurrence of Caries, Erosion, Abfraction	13. Tooth Integrity (Enamel Cracks, Tooth Fractures)	14. Periodontal Response (Always Compared to a Reference Tooth)	15. Adjacent Mucosa	16. Oral and General Health
1. Clinically very good	11.1 No hypersensitivity, normal vitality.	12.1 No secondary or primary caries.	13.1 Complete integrity.	14.1 No plaque, no inflammation, no pocket development.	15.1 Healthy mucosa adjacent to restoration.	16.1 No oral or general symptoms.
2. Clinically good	11.2 Minor hypersensitivity for a limited period of time, normal vitality. No treatment required.	12.2 Small and localized: 1. Demineralization 2. Erosion or 3. Abfraction.	13.2.1 Small marginal enamel fracture (<150 µm). 13.2.2 Hairline crack in enamel (<150 µm).	14.2 Slight plaque/inflammation (gingivitis), no pocket development. 14.2.1 Without overhangs, gaps or inadequate anatomic form. 14.2.2 With overhangs, gaps or inadequate anatomic form. Healthy after minor removal of mechanical irritations (plaque, calculus, sharp edges, etc.).	15.2 Healthy after minor removal of mechanical irritations (plaque, calculus, sharp edges, etc.).	16.2 Minor transient generalized symptoms.
3. Clinically sufficient/satisfactory	11.3.1 Moderate hypersensitivity. 11.3.2 Delayed/mild sensitivity; no subjective complaints, no treatment needed.	12.3 Larger areas of: 1. Demineralization 2. Erosion or 3. Abrasion/abfraction. Dentin not exposed. Only preventive measures necessary.	13.3.1 Marginal enamel defect < 250 µm. 13.3.2 Crack < 250 µm. 13.3.3 Enamel chipping. 13.3.4 Multiple cracks.	14.3 Difference up to one grade in severity of PBI compared to baseline and/or BOP. 14.3.1 Without overhangs, gaps or inadequate anatomic form. 14.3.2 With overhangs, gaps or inadequate anatomic form.	15.3 Alteration of mucosa but no suspicion of causal relationship with restorative material.	16.3 Transient symptoms, local and/or general.
4. Clinically unsatisfactory (but reparable)	11.4.1 Intense hypersensitivity. 11.4.2 Delayed with minor subjective symptoms. 11.4.3 No clinically detectable sensitivity. Intervention necessary but not replacement.	12.4.1 Caries with cavitation and suspected undermining caries. 12.4.2 Erosion in dentine. 12.4.3 Abrasion/abfraction in dentine. Localized and accessible; can be repaired.	13.4.1 Major marginal enamel defects; gap > 250 µm or dentine/base exposed. 13.4.2 Large cracks (probe penetrates > 250 µm). 13.4.3 Large enamel chipping or wall fracture.	14.4 Difference of more than one grade of PBI compared to control tooth or increase in pocket depth > 1 mm requiring intervention. 14.4.1 Without overhangs, gaps or inadequate anatomic form. 14.4.2 With overhangs, gaps or inadequate anatomic form.	15.4 Suspected mild allergic, lichenoid or toxic reaction. Intervention necessary but no replacement.	16.4 Persisting local or general symptoms of oral contact stomatitis, lichen planus, or allergic reactions. Intervention necessary but no replacement.
5. Clinically poor (replacement necessary)	11.5 Intense, acute pulpitis or non-vital tooth. Endodontic treatment is necessary and restoration must be replaced.	12.5 Deep caries or exposed dentine that is not accessible for restoration.	13.5 Cusp or tooth fracture.	14.5 Severe/acute gingivitis or periodontitis. 14.5.1 Without overhangs, gaps or inadequate anatomic form. 14.5.2 With overhangs, gaps or inadequate anatomic form.	15.5 Suspected severe allergic, lichenoid or toxic reaction.	16.5 Acute/severe local and/or general symptoms.

**Table A4:** Distributions of esthetic property values measured at different time points according to the etching method and type of composite used.

			Injectable Composite			CAR	Paste-Type Composite			CAR
			1.Week	6.Month	1.Year		1.Week	6.Month	1.Year	
			(1/2/3/4/5)	(1/2/3/4/5)	(1/2/3/4/5)		(1/2/3/4/5)	(1/2/3/4/5)	(1/2/3/4/5)	
Acid-Etching	Esthetic Properties	Surface Gloss	39/3/0/0/0	23/19/0/0/0	9/28/0/0/0	100%	38/4/0/0/0	21/21/0/0/0	8/30/1/0/0	100%
		Surface Staining	42/0/0/0/0	28/14/0/0/0	18/19/0/0/0	100%	42/0/0/0/0	24/18/0/0/0	15/24/0/0/0	100%
		Color Stability And Translucency	42/0/0/0/0	35/7/0/0/0	23/14/0/0/0	100%	41/1/0/0/0	36/6/0/0/0	18/21/0/0/0	100%
		Anatomic Form	42/0/0/0/0	42/0/0/0/0	37/0/0/0/0	100%	42/0/0/0/0	42/0/0/0/0	39/0/0/0/0	100%
Er,Cr:YSGG laser	Esthetic Properties	Surface Gloss	34/8/0/0/0	23/18/0/0/0	12/23/1/0/0	100%	35/7/0/0/0	24/18/0/0/0	12/24/2/0/0	100%
		Surface Staining	42/0/0/0/0	31/10/0/0/0	17/19/0/0/0	100%	42/0/0/0/0	30/12/0/0/0	19/19/0/0/0	100%
		Color Stability And Translucency	42/0/0/0/0	38/3/0/0/0	23/12/1/0/0	100%	41/1/0/0/0	35/7/0/0/0	23/15/0/0/0	100%
		Anatomic Form	42/0/0/0/0	41/0/0/0/0	36/0/0/0/0	100%	42/0/0/0/0	42/0/0/0/0	38/0/0/0/0	100%

CAR: Clinical acceptability rate.

**Table A5:** Distributions of functional and biological property scores measured at different time points according to the etching method and composite type used.

			Injectable Composite			CAR	Paste-Type Composite			CAR
			1.Week	6.Month	1.Year		1.Week	6.Month	1.Year	
			(1/2/3/4/5)	(1/2/3/4/5)	(1/2/3/4/5)		(1/2/3/4/5)	(1/2/3/4/5)	(1/2/3/4/5)	
Acid-Etching	Functional Properties	Wear	42/0/0/0/0	27/15/0/0/0	10/27/0/0/0	100%	42/0/0/0/0	26/16/0/0/0	9/30/0/0/0	100%
		Patient’s View	42/0/0/0/0	42/0/0/0/0	37/0/0/0/0	100%	42/0/0/0/0	42/0/0/0/0	39/0/0/0/0	100%
	Biological Properties	Postoperative Sensitivity	42/0/0/0/0	42/0/0/0/0	37/0/0/0/0	100%	42/0/0/0/0	42/0/0/0/0	39/0/0/0/0	100%
		Recurrence of Caries	42/0/0/0/0	42/0/0/0/0	37/0/0/0/0	100%	42/0/0/0/0	42/0/0/0/0	39/0/0/0/0	100%
		Tooth Integrity	42/0/0/0/0	42/0/0/0/0	37/0/0/0/0	100%	42/0/0/0/0	42/0/0/0/0	39/0/0/0/0	100%
		Periodontal Response	42/0/0/0/0	42/0/0/0/0	37/0/0/0/0	100%	42/0/0/0/0	42/0/0/0/0	39/0/0/0/0	100%
		Adjacent Mucosa	42/0/0/0/0	42/0/0/0/0	37/0/0/0/0	100%	42/0/0/0/0	42/0/0/0/0	39/0/0/0/0	100%
		Oral and General Health	42/0/0/0/0	42/0/0/0/0	37/0/0/0/0	100%	42/0/0/0/0	42/0/0/0/0	39/0/0/0/0	100%
Er,Cr:YSGG laser	Functional Properties	Wear	42/0/0/0/0	30/11/0/0/0	17/19/0/0/0	100%	42/0/0/0/0	25/17/0/0/0	15/23/0/0/0	100%
		Patient’s View	42/0/0/0/0	41/0/0/0/0	36/0/0/0/0	100%	42/0/0/0/0	42/0/0/0/0	38/0/0/0/0	100%
	Biological Properties	Postoperative Sensitivity	36/6/0/0/0	41/0/0/0/0	36/0/0/0/0	100%	34/8/0/0/0	42/0/0/0/0	38/0/0/0/0	100%
		Recurrence of Caries	42/0/0/0/0	41/0/0/0/0	36/0/0/0/0	100%	42/0/0/0/0	42/0/0/0/0	38/0/0/0/0	100%
		Tooth Integrity	42/0/0/0/0	41/0/0/0/0	36/0/0/0/0	100%	42/0/0/0/0	42/0/0/0/0	38/0/0/0/0	100%
		Periodontal Response	42/0/0/0/0	41/0/0/0/0	36/0/0/0/0	100%	42/0/0/0/0	42/0/0/0/0	38/0/0/0/0	100%
		Adjacent Mucosa	42/0/0/0/0	41/0/0/0/0	36/0/0/0/0	100%	42/0/0/0/0	42/0/0/0/0	38/0/0/0/0	100%
		Oral and General Health	42/0/0/0/0	41/0/0/0/0	36/0/0/0/0	100%	42/0/0/0/0	42/0/0/0/0	38/0/0/0/0	100%

CAR: Clinical acceptability rate.
